# Lipidoid Nanoparticles for siRNA Delivery to the Intestinal Epithelium: In Vitro Investigations in a Caco-2 Model

**DOI:** 10.1371/journal.pone.0133154

**Published:** 2015-07-20

**Authors:** Rebecca L. Ball, Christopher M. Knapp, Kathryn A. Whitehead

**Affiliations:** 1 Department of Chemical Engineering, Carnegie Mellon University, Pittsburgh, Pennsylvania, United States of America; 2 Department of Biomedical Engineering, Carnegie Mellon University, Pittsburgh, Pennsylvania, United States of America; Emory University School of Medicine, UNITED STATES

## Abstract

Short interfering ribonucleic acid (siRNA) therapeutics show promise for the treatment of intestinal diseases by specifically suppressing the expression of disease relevant proteins. Recently, a class of lipid-like materials termed “lipidoids” have been shown to potently deliver siRNA to the liver and immune cells. Here, we seek to establish the utility of lipidoid nanoparticles (LNPs) in the context of siRNA delivery to the intestinal epithelium. Initial studies demonstrated that the siRNA-loaded LNPs mediated potent, dose dependent, and durable gene silencing in Caco-2 intestinal epithelial cells, with a single 10 nM dose depressing GAPDH mRNA expression for one week. Transfection with siRNA-loaded LNPs did not induce significant cytotoxicity in Caco-2 cells or alter intestinal barrier function. Protein silencing was confirmed by Western blotting, with the lowest levels of GAPDH protein expression observed five days post-transfection. Together, these data underscore the potential of LNPs for the treatment of intestinal disorders.

## Introduction

Intestinal diseases such as gastrointestinal cancer and inflammatory bowel disease (IBD) adversely affect the health and quality of life of millions of people worldwide[1,2]. Although numerous factors such as genetics, microbiota composition, environment and the immune system have been shown to play a specific role in development of these diseases, the underlying causal biology is complex and not fully understood[1,3–7]. However, there are some instances in which diseases of the gut have been associated with an over-expression of one or more proteins[6,8–10]. Accordingly, such intestinal maladies could potentially be treated with RNA interference (RNAi) theraputics, which rely on short interfering RNA (siRNA) to transiently knockdown the expression of problematic genes.

As with the rest of the RNAi space, one of the key challenges in developing a clinically viable therapy for the intestines is the identification of a stable siRNA delivery vehicle that achieves potent gene silencing without inducing toxicity or immune stimulation[11,12]. To date, there has been limited work done in this area. Some classes of nanoparticles, including polymers and lipids, have been shown to deliver various drugs, including aminosalicylates, corticosteriods, and immunosuppresives, to intestinal epithelial cells in vitro and in vivo[12–14]. Other studies have shown that several types of nanoparticles and microspheres can be used to deliver siRNA to the macrophages within the gastrointestinal (GI) tract [15–17]. The most common gene target studied has been the inflammatory cytokine, TNF-α, which is produced by macrophages and has been implicated in the progression of inflammatory bowel disease[4,18]. Although anti-TNF-α therapies can be effective in alieviating symptoms of IBD in some patients, serious side effects associated with continual immunosuppressive therapy often limits their use in the clinic [19,20].

Little focus has been placed on gene silencing within the epithelial cells that provide barrier function within the small and large intestines. In one study, amphiphilic polyallylamine polymeric micelles were shown to facilitate moderate levels of siRNA delivery to intestinal cells in vitro[21]. We believe delivery to epithelial cells warrants greater attention, as this approach may have utility in the treatment of several intestinal maladies. For example, intestinal cells prone to polyp formation have been shown to upregulate beta-catenin [22,23]. Another condition that may benefit from gene silencing therapy is salmonella infection, as salmonella bacteria mediate an upregulation in the tight junction protein claudin-2, leading to a leaky intestinal barrier[24].

Our approach seeks to use degradable lipidoid nanoparticles (LNPs) for siRNA delivery to the intestinal epithelium. The active delivery conponents of LNPs are lipid-like molecules, termed lipidoids, that are synthesized by the Michael addition of alkyl-amines to alkyl-acrylates[25]. Degradable LNPs have previously been shown to mediate potent gene knockdown in a variety of cell targets, including hepatocytes and immune cells, upon IV administration to mice[26]. Furthermore, they did not induce toxicity or immune stimulation as assessed by liver histology, hematological analysis, or cytokine profiling. Given the critical need for effective intestinal siRNA delivery systems, we have asked whether or not lipidoid nanoparticles may be amenable for use as intestinal disease therapeutics. Herein, we demonstrate proof-of-principle that LNPs can potently and durably induce gene and protein silencing in an established in vitro model of the intestinal epithelium without provoking cytotoxicity or loss of barrier function.

## Materials and Methods

### Materials

Cholesterol was purchased from Sigma Aldrich, while distearoyl-sn-glycerol-3-phosphocholine (DSPC) and PEG2000-DMG were obtained from Avanti Polar Lipids. Caco-2 cells were purchased from the American Type Culture Collection. Minimum Essential Media (MEM), trypsin, penicillin/streptomyocin, phosphate buffered saline (PBS), and fetal bovine serum (FBS) were purchased from Life Technologies. Antibodies reactive to glyceraldehyde 3-phosphate dehydrogenase (GAPDH) and alpha tubulin were purchased from Abcam. Silencer Select siRNA for GAPDH and green flourescence protein (GFP) were orderd from Life Technologies.

### LNP formulation

LNPs were prepared as previously described[26]. Briefly, lipidoids, cholesterol, DSPC, PEG2000-DMG and siRNA were dissolved in ethanol and mixed at a molar ratio of 50:38.5:10:1.5 in a solution of 90% ethanol and 10% 10 nM sodium citrate (by volume). An siRNA solution was prepared by diluting siRNA in 10 nM sodium citrate such that the final weight ratio of lipidoid: siRNA was 5:1. Particles were formed upon rapid pipet mixing of equal volumes of siRNA solution, and the particles were diluted in PBS.

### Nanoparticle characterization

The LNPs were diluted to an siRNA concentration of 1 μg/ ml in PBS buffer, pH 7.4 for all charcterization studies. The siRNA entrapment efficiency was determined using Quanti-iT Ribogreen assay (Invitrogen) following manufacturer’s protocol. LNP particle size and zeta potential were measured with a Malvern Zetasizer Nano (Malvern Instruments, UK).

### Cell culture

Caco-2 cells were purchased from the American Type Culture Collection (ATCC) and grown in MEM supplemented with 200 ml/L of FBS, 10 IU/ml of penicillin, and 10 mg/l streptomyocin. The cells were kept at 37°C in a 5% CO_2_ environment. The cells were subcultured by partial digestion with 0.25% trypsin and EDTA. Passages 25–60 were used for experiements. For monolayer experiments, Caco-2 cells were grown on Corning BioCoaT HTS 1.0 μm filter support transwell plaes in Basal Seeding Medium (BSM) for 2 days and Entero-STIM Enterocyte Differentiation Medium (EDM) for 1 day. Both the BSM and EDM were supplmented with MITO+ Serum Extender according to the manufacturer’s protocol. The transepithelial electrical resistance (TEER) was measured with a Millicell Voltohmmeter to confirm the existance of a viable monolayer. Only Caco-2 monolayers with TEER values above 300 Ωcm^2^ were used for experiments.

### Methyl thiazole tetrazolium (MTT) experiments

Caco-2 cells were seeded at 10^4^ cells/well in 96 well plates. The LNPs were incubated with the cells for 24 hours, after which 10 μl of MTT reagent was applied to each well. After 3 hours, 100 μl of detergent was applied to each well and incubated in the dark overnight at room temperature. The absorbance was read at 570 nm and sample values were normalized to untreated controls.

### RNA isolation and reverse transcription

Caco-2 cells were seeded onto 6-well plates at a density of 5 x 10^5^ cells/well. The total RNA was isolated using Qiagen RNeasy Kit according to the manufacturer’s protocal. The total RNA concentration was determined by absorbance at 260/280 nm using a Nanodrop 2000 UV-Vis spectrophotometer (Thermo Scientific). Reverse transcriptase PCR was carried out using the high capacity cDNA reverse transcription kit (Applied Biosystems) according to the manufacturer’s protocol.

### Quantification of gene expression using quantitative PCR

The quantitative PCR (qPCR) was carried out using the ViiA 7 Real-Time PCR system and Taqman universal PCR master mix (Applied Biosystems). Each quantitative PCR reaction contained a total volume of 20 μl (100 ng cDNA + 10 μl Taqman mastermix + 1 μl Tagman endogenous control + 1 μl Taqman gene expression). The primers/probes for GAPDH (Hs02758991_g1) and beta actin (Hs01060665_g1) were ordered from Life Technologies using the best coverage primer/ probe set. All runs were performed in comparative Ct mode with temperatures at 50°C for 2 minutes, 95°C for 10 minutes, 40 cycles of 95°C for 15 seconds and 60°C for 1 minute. All qPCR samples were tested in biological and technical triplicates. The expression of GAPDH mRNA was normalized with beta-actin mRNA expression and presented relative to the control sample GAPDH mRNA.

### Detection of GAPDH by Western Blot

The protein expression of GAPDH was analyzed by Western Blot. Caco-2 cells (5 x 10^5^) were seeded on a 6-well plate and grown for desired time periods. Cells were washed with ice-cold PBS and lysed with a lysis buffer consisting of 50nM Tris HCL, 150mM NaCl, 2nM EDTA, 1.0% Triton X-100, and mammalian protease inhibitor cocktail (Amresco). The cells were then scraped and transferred to a precooled microcentrifuge tube. The samples were maintained on ice with constant agitation for 30 minutes, vortexing for 10 sec every 10 minutes. The samples were then centrifuged for 10 minutes at 14,000 rpm and the supernatant was collected for further use. The total protein concentration of each sample was determined using a Bicinchoninic acid assay from Pierce. 20 μg of protein from each sample was loaded into a mini 4–15% SDS-PAGE (TGX Mini-Protean BioRad). The gel was dry blotted onto a PVDF membrane using an iBlot system (Life Technologies). Immunodetection was performed using the WesternBreeze Chemiluminescent Kit (Thermo Fisher Scientific). Primary GAPDH and alpha-tubulin antibodies were purchased from Abcam. Chemiluminescence detection was performed on the PVDF membrane using the ImageQuant LAS 400 (GE Healthcare). The relative densities/ intensities of the detected protein bands were analyzed with ImageJ.

## Results

### Formulation and characterization of siRNA LNPs

**Fig 1:**
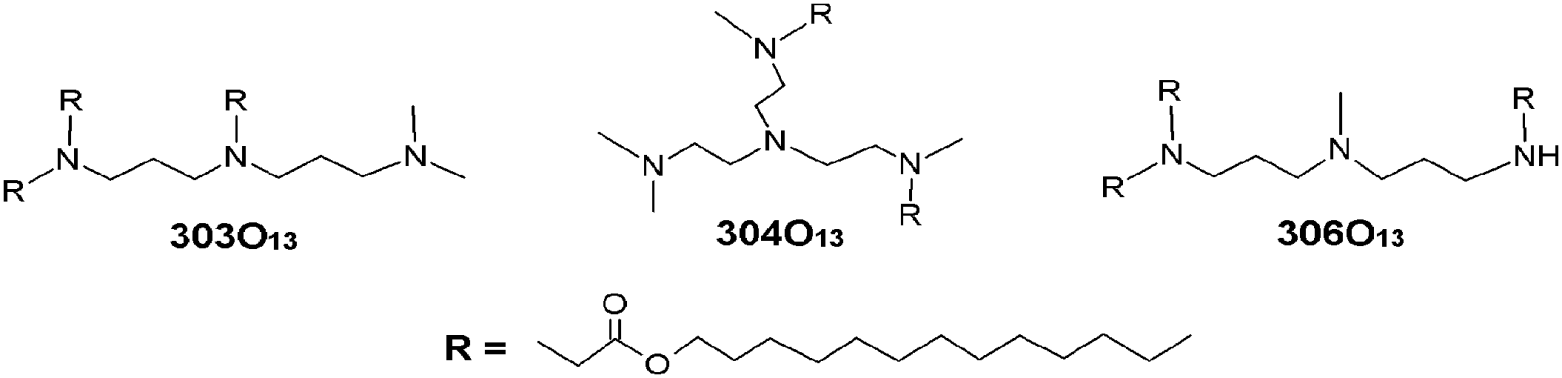
The three lipidoids used in this study were synthesized by the Michael addition of alkyl-acrylates to alkyl-amines.

Three chemically distinct lipidoids (Fig 1) were evaluated for their ability to deliver siRNA to Caco-2 intestinal epithelial cells. These lipidoids were chosen because they have been shown previously to mediate potent gene silencing in several cell and organ types in vitro and in vivo [26]. Tertiary amines, in particular, have been found to confer transfection efficacy both for lipidoid nanoparticles and other siRNA delivery systems[27–29]. We began our studies by comparing the silencing efficacy of these LNPs on intestinal epithelial cells (Caco-2 cells) in order to select the most potent for future experiments.

**Fig 2:**
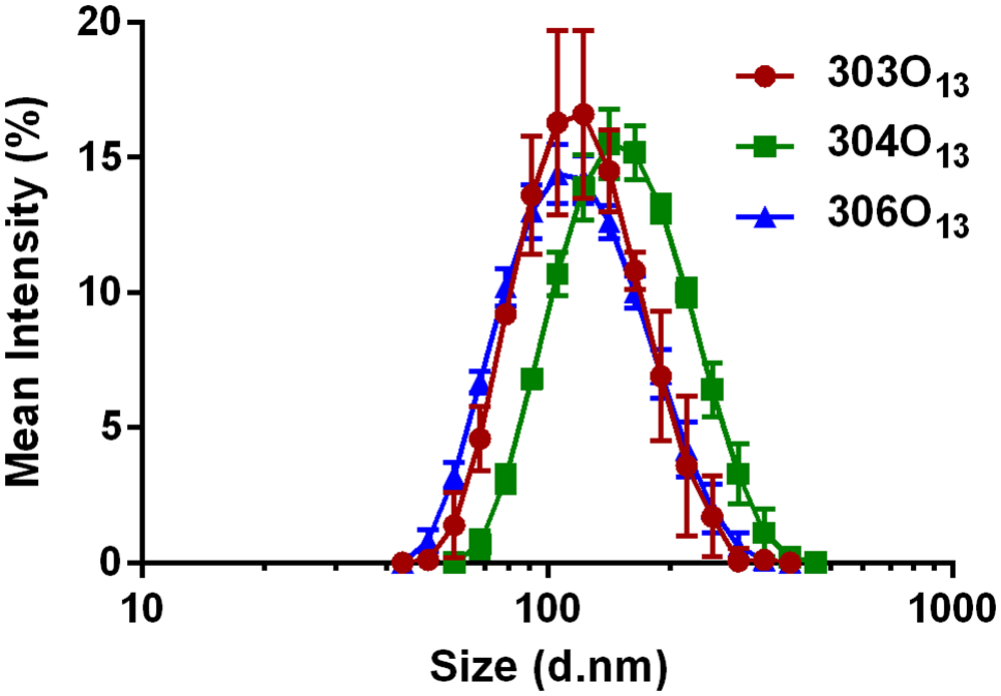
The three LNPs had average diameters on the order of 100 nm as determined by dynamic light scattering. The mean light intensity is shown vs. diameter size distribution for each lipidoid nanoparticle. Error bars represent s.d. (n = 3).

LNPs were characterized prior to experimentation, and the data is assembled in Table 1. A Quant-IT Ribogreen Assay determined the siRNA entrapment (or loading efficiency) to be ~90% for each of the three LNPs (Table 1). In general, it has been found that entrapment values of 75% or higher to correspond to the most potent gene silencing in vivo[30]. Furthermore, LNP size was measured using dynamic light scattering (DLS), with histograms of the intensity size distribution shown in Fig 2. LNP z-average diameter size ranged from approximately 110 nm to 145 nm with polydispersity indices below 0.2. Additionally, the zeta potential of the three different LNPs was found to be slightly negative at pH ~ 7.4.

**Table 1:** LNP characterization data.

LNP	Size (nm)	PDI*	Zeta Potential (mV)	siRNA Entrapment (%)
303O_13_	120 ± 3	0.18	-11 ± 0.78	93.5 ± 0.4
304O_13_	144 ± 2	0.10	-3.52 ± 0.32	90.4 ± 0.7
306O_13_	111 ± 1	0.15	-10.8 ± 0.25	93.8 ± 0.5

Size and polydispersity index (PDI) were determined by dynamic light scattering with histograms shown in Fig 2. Zeta potential was measured at a pH of 7.4. Size values represent the z-average diameter. Size, zeta potential and siRNA entrapment values represent the mean ± s.d. (n = 3).

*PDI: Polydispersity index

### Gene silencing in undifferentiated Caco-2 cells

**Fig 3:**
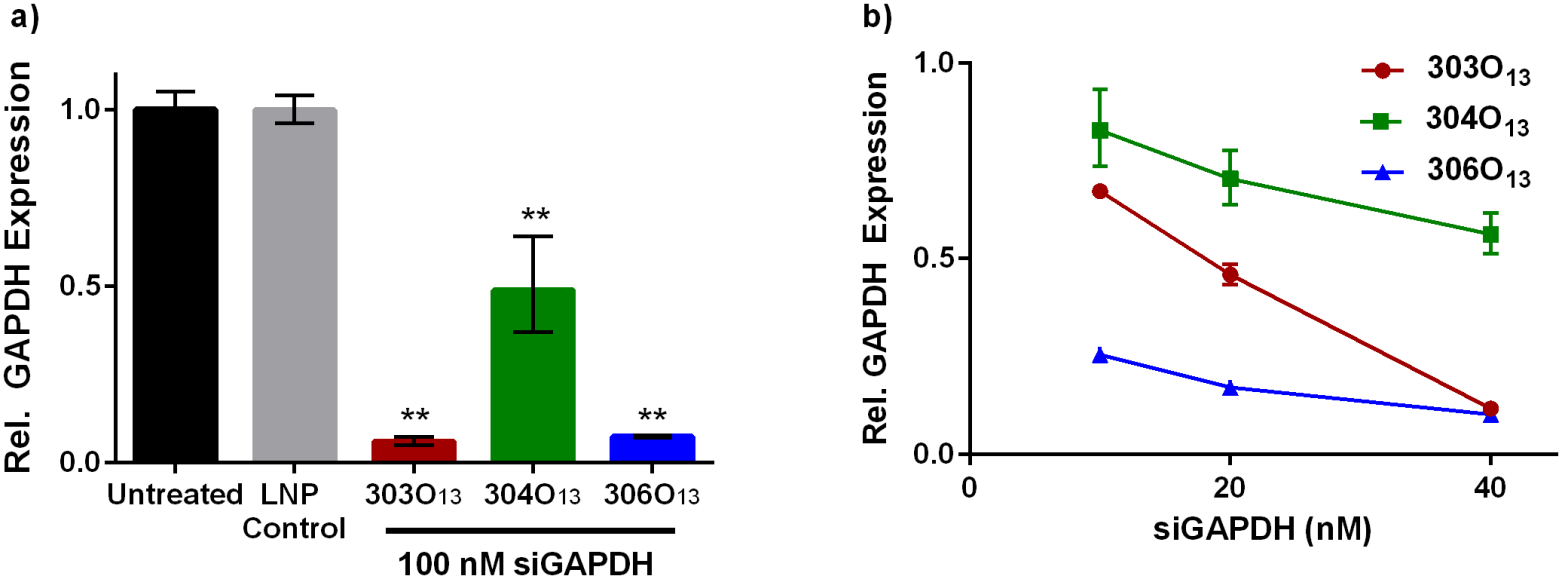
All three LNPs facilitated GAPDH mRNA silencing in Caco-2 cells. **A)** LNPs 306O_13_ and 303O_13_ mediated ~90% silencing at an siRNA dose of 100 nM while control LNPs containing off-targeted siRNA did not induce statistically significant changes in gene expression. ** p < 0.001 as determined by an unpaired student’s t-test to the untreated control. **B)** The 306O_13_ LNPs had the most potent dose-dependent GAPDH gene silencing in Caco-2 cells. The LNP control consisted of 100 nM siGFP 306O_13_ LNPs. Error bars represent s.d. (n = 3).

LNPs were then evaluated for their capacity to induce gene silencing in intestinal epithelial cells. In this study, we chose to work with Caco-2 cells, which are human colorectal adenocarcinoma cells commonly used to model the intestinal epithelium [31,32]. Upon differentiation, Caco-2 cells polarize into monolayers expressing microvilli and tight junctions that serve as a barrier to absorption [31–33]. For this proof-of-concept analysis, we sought to silence the model “housekeeping” protein, GAPDH, which is typically expressed at high levels and can be knocked down without causing adverse cellular events [34,35]. LNPs were delivered at an siGAPDH concentration of 100 nM to undifferentiated Caco-2 cells, and GAPDH mRNA expression was measured by qPCR 24 hours post-transfection. At this dose, 306O_13_ and 303O_13_ LNPs mediated high levels of GAPDH silencing of around 90% (Fig 3A). In order to identify the best lipidoid for Caco-2 work between the two, dose response experiments were conducted (Fig 3B). Results illustrate that 306O_13_ (blue curve with triangles) facilitated the most potent dose dependent response, with 75% GAPDH silencing achieved at a low dose of 10 nM. Based on this gene silencing data, the lipidoid 306O_13_ was used in all further gene silencing experiments.

### Cytotoxicity

**Fig 4:**
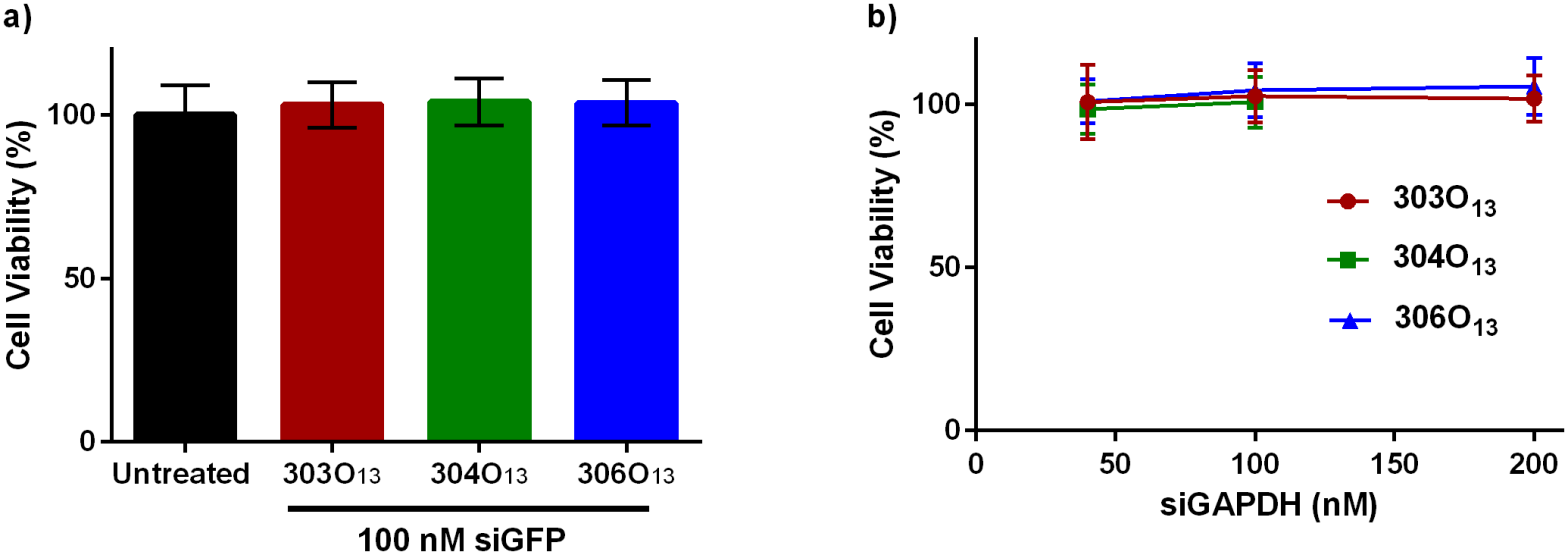
Neither A) Control (siGFP) nor B) siGAPDH loaded LNPs adversely affected Caco-2 cell viability at siRNA doses up to 200 nM. All LNPs were incubated with cells for 24 hours prior to measuring viability with an MTT assay. Error bars represent s.d. (n = 7).

An MTT assay was performed to assess potential LNP cytotoxicity. In these experiments, LNPs containing siGAPDH or control siGFP at three siRNA doses were incubated with Caco-2 cells for a period of 24 hours. As shown in Fig 4, none of the LNPs significantly affected cell viability compared to untreated cells as determined by an unpaired student’s t-test with a 95% confidence interval. These experiments suggest that neither the LNP delivery vehicles nor the act of silencing GAPDH cause cell toxicity at a 24 hour time point.

### Time study of GAPDH gene silencing in Caco-2 cells

**Fig 5:**
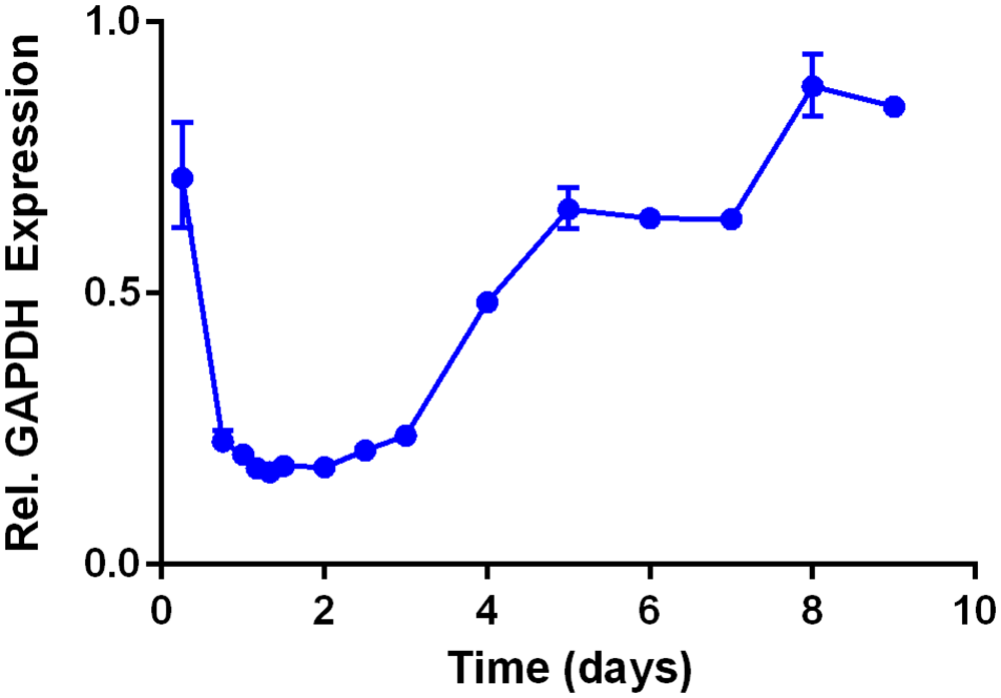
GAPDH silencing in Caco-2 cells with the LNP 306O_13_ was potent and durable. A single dose of 10 nM siGAPDH depressed mRNA expression for one week, with maximal silencing of 80% observed 24–60 hours post-transfection. Error bars represent s.d. (n = 3).

A time course study was then performed in which GAPDH mRNA expression was measured at 15 time points following a single 10 nM dose of siRNA in 306O_13_ LNPs. The data in Fig 5 indicate that maximum knockdown at the mRNA level of >80% occurred 24–60 hours after LNP transfection. In addition to being potent, gene silencing was also quite durable, with a 10 nM siRNA dose depressing gene expression for approximately one week.

### 306O_13_ mediated gene silencing in Caco-2 cell monolayers

**Fig 6:**
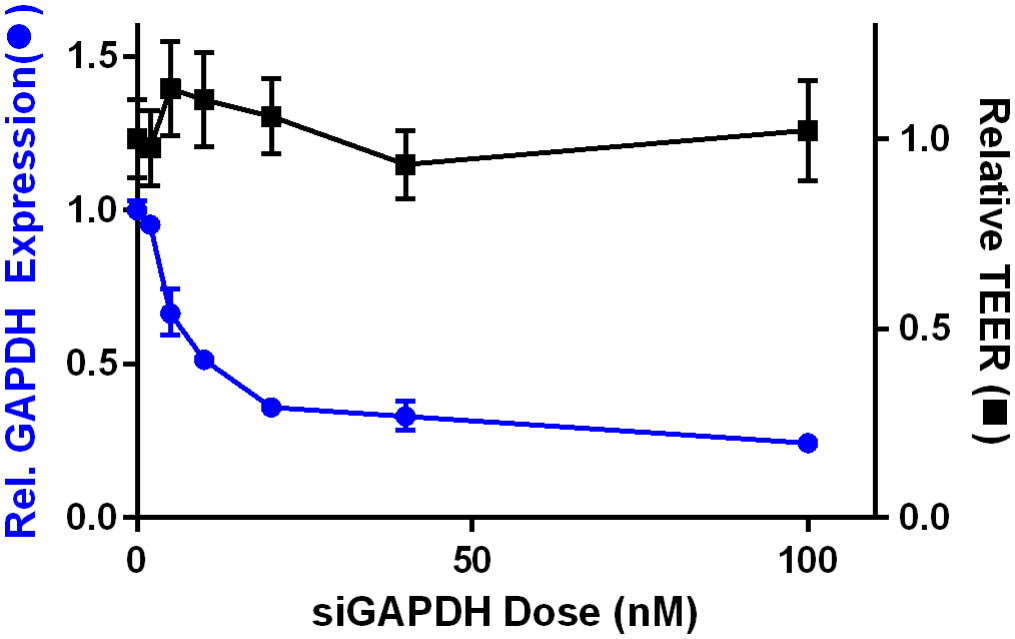
The LNP 306O_13_ facilitated dose dependent GAPDH mRNA silencing in Caco-2 monolayers 24 hours post-transfection. Caco-2 monolayer barrier function as measured by TEER was not affected by LNP mediated gene silencing. Dose response data (blue circles) indicate an EC_50_ siGAPDH dose of 10 nM. TEER values (black squares) are reported 24 hours post-transfection relative to the time of transfection (t = 0), normalized to untreated cells. Error bars represent s.d. (n = 3–6).

We next sought to confirm that similar levels of gene knockdown could be obtained using LNPs on Caco-2 cells monolayers compared to the results for undifferentiated Caco-2 cells described above. In these experiments, fully-formed Caco-2 monolayers were incubated with siGAPDH-loaded 306O_13_ LNPs for 24 hours. As shown by the blue curve in Fig 6, a dose dependent response was observed, with the most potent GAPDH gene silencing of ~76% occurring with an siRNA dose of 100 nM. The EC_50_ dose for the Caco-2 monolayers was approximately 10 nM. Monolayer integrity was monitored for each dose throughout the experiment via transepithelial electric resistance (TEER) measurements. TEER has been demonstrated to correlate inversely with epithelial permeability to small molecules[36]. The TEER data points shown in Fig 6 (black curve) represent TEER values 24 hours post transfection relative to initial TEER values at the time of transfection. The TEER data shown in Fig 6 suggest that incubation with LNPs does not induce significant changes to epithelial barrier function during 24 hours of exposure for any of the six doses tested. The LNP control which consisted of 306O_13_ with siGFP did not significantly alter the relative GAPDH gene expression as well as the TEER when compared to untreated cells.

### LNP effect on GAPDH protein expression

**Fig 7:**
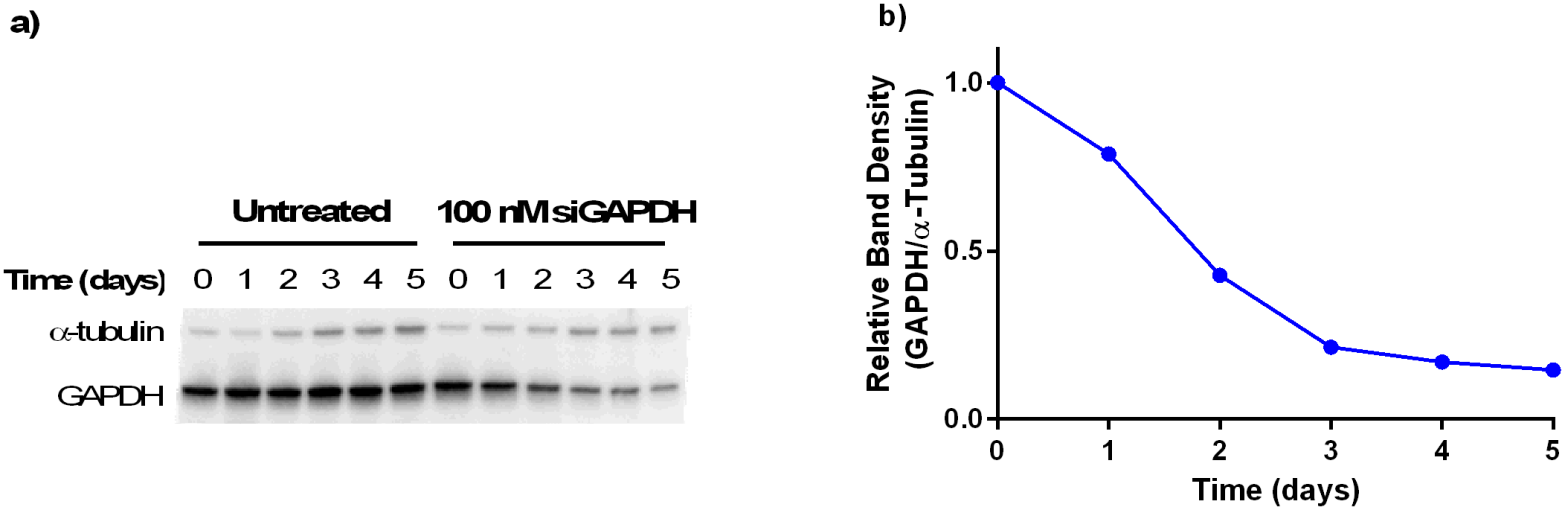
A single dose of 100 nM siGAPDH 306O_13_ LNPs gradually reduced GAPDH protein silencing in Caco-2 cells over a period of 5 days. **a)** α-tubulin was used as a loading control and blot shows chemiluminescence signal from a PVDF membrane. **b)** Plotting the band density of GAPDH relative to α-tubulin, as quantified by ImageJ, suggests that a high level of protein silencing 85% was achieved by Day 5.

Finally, a western blot analysis determined the effect of the 100 nM siRNA dose of 306O_13_ siGAPDH LNPs on GAPDH protein expression. For this experiment, protein lysate was collected every day for five days post-transfection. As seen in Fig 7, the amount of GAPDH protein began to decrease around 1 or 2 days post transfection. Alpha-tubulin was used as a protein loading control for comparison of the GAPDH protein expression over time. Band quantification using ImageJ suggested that the siRNA-loaded LNPs could reduce the protein expression of GAPDH by approximately 85% five days post-transfection.

## Discussion

Intestinal diseases such as IBD and gastrointestinal cancer are associated with damaging symptoms and ineffective treatments. Presently, a number of proteins have been identified that are upregulated in these intestinal maladies and may be amenable to RNAi therapy. Unfortunately, siRNA delivery has not been straightforward, in part due to its relatively large size (~13 kDa) and its overall negative charge attributable to its phosphate backbone[11]. A major challenge of cellular siRNA delivery is endosomal escape and entry into the cytoplasm, which is where siRNA can load into the RISC complex enroute to cleaving the target mRNA[23]. Tertiary amine groups that contain a hydrophobic chain, similar to the lipidoid structure, have been hypothesized to use the proton sponge effect in order to escape the endosome. The tertiary amine groups have a high buffer capacity and upon protonation in the endosome become detergents that disrupt the membrane of the endosome[37]. The LNPs could potentially be affecting the endosomal membrane in this way leading to the release of the siRNA into the cytoplasm and potent gene silencing at lower siRNA doses.

Nanoscale delivery vehicles have been shown to have improved cellular uptake when compared to delivery vehicles on the microscale[38]. Previous research has shown that nanoparticles approximately 100 nm or less are able to diffuse smoothly through the mucus layer covering the intestinal cells; however, other research has reported that nanoparticles 500 nm in diameter are able to cross the mucus barrier and become absorbed by enterocytes[1,39]. The diameters of the three LNPs examined in this study (111 nm– 140 nm) suggest that they may be appropriate for in vivo delivery to the intestinal epithelium.

Some cationic delivery vehicles have been shown to induce toxic effects at effective gene silencing doses due to harmful positive charge interactions with the cell membrane [21,40]. The degradable lipidoid nanoparticles used in this study had a slightly negative charge at neutral pH and did not significantly induce cytotoxicity in Caco-2 cells, even at fairly high concentration of 200 nM. Lipidoids take on a positive charge only under reduced pH conditions (e.g. in the endosome), with the surface pKa of 306O_13_ nanoparticles reported to be approximately 6.8[26]. Non-toxic therapeutic delivery systems may be particularly important for the treatment of intestinal diseases where continuous treatment may be required due to the high turnover rate of intestinal cells as well as the frequency of disease relapses[7,41].

Of the three LNPs studied, 306O_13_ most potently silenced GAPDH expression in undifferentiated Caco-2 cells, with a 100 nM siRNA inducing ~90% target gene silencing after 24 hours. When transfecting Caco-2 cells with a low dose of 10 nM siGAPDH LNPs, the highest silencing was seen 24–60 hours post transfection. If desired, such data would inform a dosing regimen capable of suppressing gene silencing for an extended period of time. For example, as seen in Fig 5, the LNPs would need to be delivered every three days at a dose of 10 nM in order to maintain maximal GAPDH silencing. It is likely that higher doses would allow a longer duration between doses. Because the dynamics of expression upon silencing are specific to each unique gene, such an experiment would need to be run for the gene of interest in order to develop a therapeutic dosing regimen. Western blotting experiments showed that GAPDH down regulation at the protein level lagged behind mRNA down regulation by approximately 4 days [42]. Gene silencing following a single 10 nM dose of siRNA was surprisingly durable, with GAPDH mRNA expression depressed for approximately one week. In general, healthy intestinal cells have a high turnover rate of 1–2 days and an increase in epithelial cell proliferation is seen in patients with IBD and gastrointestinal cancer [41,43].

LNPs were slightly less efficacious when transfecting differentiated Caco-2 monolayers (76% GAPDH silencing) compared to undifferentiated cells (92%) at an siRNA dose of 100 nM. The decrease in gene silencing potency could be due to the columnar form of the differentiated epithelial cells, which decreases the cell surface area available for LNP uptake. Furthermore, monolayers express microvilli upon differentiation, and LNPs need to navigate into the intermicrovillar space in order for endocytosis to occur [31,44].

When developing epithelial delivery systems, it is important to account for the vehicle’s effect on the permeability of the epithelium. An increase in permeability could lead to the vehicle distributing to undesirable places (systemic) or exacerbating the intestinal disease. This is particularly relevant when considering the treatment of several gastrointestinal disorders, as increased tight junction permeability has been implicated in irritable bowel syndrome, IBD, and celiac disease[1,8,45]. Importantly, the delivery of LNPs to Caco-2 monolayers did not alter the tight junction permeability as determined by TEER measurements. Importantly, it should be noted that TEER measures the resistance of the ion flux through the tight junctions, and caution should be used in extrapolating results to the permeability of drugs[46,47].

Previous animal studies utilizing the siRNA-loaded LNPs have shown their ability to mediate potent gene knockdown in a variety of cell targets, including hepatocytes and immune cells, upon IV administration to mice[26]. Furthermore, they did not induce toxicity or immune stimulation as assessed by liver histology, hematological analysis, or cytokine profiling. The previous results and the data represented in this manuscript show promise that the LNPs will perform well in vivo when targeting intestinal gene expression. Moreover, the LNPs have been shown to compete if not perform better than the comerical siRNA delivery reagent, Lipofectamine2000, when silencing luciferase in HeLa cells[48].

Overall, lipidoid nanoparticles show promise as siRNA delivery vehicles for intestinal disease therapeutics as a result of their high siRNA entrapment efficiency and their ability to potently and durably induce gene silencing in Caco-2 intestinal epithelial cells. Essentially, this low-dose gene silencing can be accomplished without compromising intestinal barrier function or inducing cytotoxicity. This study serves as a proof of concept that LNPs can facilitate robust gene silencing in the intestinal epithelium, and future work will focus on the silencing of therapeutically relevant proteins that have been implicated in intestinal disease.
